# Differentiation of Organic Cocoa Beans and Conventional Ones by Using Handheld NIR Spectroscopy and Multivariate Classification Techniques

**DOI:** 10.1155/2021/1844675

**Published:** 2021-11-20

**Authors:** Elliot K. Anyidoho, Ernest Teye, Robert Agbemafle

**Affiliations:** ^1^University of Cape Coast, College of Agriculture and Natural Sciences, School of Agriculture, Department of Agricultural Engineering, Cape Coast, Ghana; ^2^Ghana Cocoa Board, Cocoa Health and Extension Division, Elubo, Ghana; ^3^University of Cape Coast, Africa Centre of Excellence for Food Fraud and Safety Food, AfriFoodinTegrity Centre, Cape Coast, Ghana; ^4^University of Cape Coast, College of Agriculture and Natural Sciences, School of Physical Sciences, Department of Laboratory Technology, Cape Coast, Ghana

## Abstract

The global market for organic cocoa beans continues to show sturdy growth. A low-cost handheld NIR spectrometer (900-1700 nm) combined with multivariate classification algorithms was used for rapid differentiation analysis of organic cocoa beans' integrity. In this research, organic and conventionally cultivated cocoa beans were collected from different locations in Ghana and scanned nondestructively with a handheld spectrometer. Different preprocessing treatments were employed. Principal component analysis (PCA) and classification analysis, RF (random forest), KNN (*K*-nearest neighbours), LDA (linear discriminant analysis), and PLS-DA (partial least squares-discriminant analysis) were performed comparatively to build classification models. The performance of the models was evaluated by accuracy, specificity, sensitivity, and efficiency. Second derivative preprocessing together with PLS-DA algorithm was superior to the rest of the algorithms with a classification accuracy of 100.00% in both the calibration set and prediction set. Second derivative algorithm was found to be the best preprocessing tool. The identification rates for the calibration set and prediction set were 96.15% and 98.08%, respectively, for RF, 91.35% and 92.31% for KNN, and 90.38% and 98.08% for LDA. Generally, the results showed that a handheld NIR spectrometer coupled with an appropriate multivariate algorithm could be used in situ for the differentiation of organic cocoa beans from conventional ones to ensure food integrity along the cocoa bean value chain.

## 1. Introduction

Several modern-day environmental challenges are rooted in agri-food schemes. These schemes are held partly accountable for the decrease in ecosystem destruction, water pollution, global warming, and biodiversity. Hence, the greening of agri-food production, processing, and marketing can be an important contribution to quality, safety, and sustainability. The advent of post-Fordism has put environmental issues and quality matters at the heart of agri-food provisioning schemes [1, 2].

The enhancement of sustainability performance in the cocoa industry is developing as a strategy within universal product value chains. In making the global cocoa chain and network sustainable, both private and public players have introduced many initiatives at different levels. The main driver of this trend is the emerging consumer demand for socially fair and eco-friendly products. For instance, sales of organic chocolate reached USA $304 million in 2005, representing an increase of 75% in comparison to 2002 sales [3]. Much attention has to be shifted to West Africa because it produces more than 70% of all cocoa and is the location of many organic initiatives. Ghana the second largest exporter of cocoa started the exportation of organic cocoa in 2005 to the global market. More than 20,000 smallholder farmers are currently involved in the organic cocoa network, as well as other stakeholders at national levels, such as nongovernmental organizations, farmers' organizations, several public institutions, licensed buying companies, and importers [4]. Inferentially, the most important bean category which influences and drives consumers' preference, nutritional composition, quality, and safety is the organic cocoa bean category [5]. Organic cocoa beans unlike conventional ones are cocoa beans produced following the farming practices and principles that do not allow the use of growth-stimulating elements, herbicides, synthetic pesticides, and fertilizers [5, 6]. Concerns about growth-stimulating elements, herbicides, synthetic pesticides, and fertilizers have given additional motivation to organic cocoa bean demand, as consumers progressively query the quality and safety of conventional cocoa beans [7]. Relative to the aforementioned factors, the demand for organic cocoa beans by chocolate producers and consumers has increased, and the production of organic cocoa beans is more lucrative due to the higher price it receives [8]. The higher price for the organic label as compared to the conventional cocoa beans has led to mislabeling which is regarded as fraud to gain undeserved economic advantage. The international market and consumers, thus, call for trust tags for organically produced cocoa beans [9, 10]. Therefore, screening of organic cocoa beans before export, marketing, and processing to prevent mislabeling has become very necessary.

Currently, the techniques for ensuring the integrity and quality of organic cocoa beans are mostly cumbersome, time-consuming, expensive, involve destructive means, and require highly skilled personnel and are often not applicable in low-resource countries. The use of handheld NIR spectrometer and chemometric analysis for ensuring the integrity and authenticity of organic cocoa beans from conventional ones could provide a big help. This would offer a rapid, nondestructive, and less expensive technique for the assessment of organic and conventional cocoa beans for quality control and assurance purposes.

Near-infrared (NIR) spectroscopy technique has become increasingly significant among other established green advance techniques in food technology. It provides a nondestructive analytical tool, more especially for the assessment of chemical composition and physical quality characteristics of cocoa bean and cocoa products [11]. This is due to its sensitivity to OH, CH, and NH absorptions associated with cocoa bean components. It is fast, requires little or no sample preparation, has low operating cost, and is environmentally friendly [12]. In other studies, the NIR spectroscopy has been used for the quantification of moisture content, nitrogen, and fat of cocoa powder [13], prediction of procyanidins in cocoa [14], differentiation of Ghana cocoa beans and cocoa bean varieties [15, 16], verification of cocoa powder authenticity [17], classification and determination of chemical quality parameters [18–20], and estimation of cocoa bean parameters [21]. A critical study of recent applications of the use of NIR spectroscopic technique in the cocoa bean industry showed that it has also been applied in the rapid detection of cocoa bean adulterations and fraud [22, 23] and quality control of commercial cocoa beans [24]. Therefore, NIR spectroscopy offers a reliable alternative for the assessment of organic cocoa bean integrity and quality.

Additionally, advancement in NIR instrumentation has led to the miniaturization of stationary laboratory-based NIR spectrometers into lightweight handheld spectroscopic instruments that are simple, relatively less expensive, reliable, and provide extra speed. Their portability makes them ideal instruments for *in situ* assessments of agricultural products. In this regard, the cocoa bean industry is expected to benefit from the current interest in miniaturizing NIR spectroscopic technology. However, no studies have investigated the application of handheld NIR spectrometer for screening and ensuring the integrity of organic cocoa beans nondestructively. Also, no information is available on the application of different multivariate classification algorithms for effective and accurate discrimination of organic cocoa beans.

Therefore, the objective of this work was to use a handheld NIR spectrometer and multivariate classification techniques to nondestructively identify organic cocoa beans from conventional cocoa beans. Specifically, the study is aimed at determining the ideal multivariate classification algorithm for the accurate differentiation of organic and conventional cocoa beans.

## 2. Materials and Methods

### 2.1. Cocoa Bean Samples

A total of 120 organic cocoa bean samples ready for exportation were obtained from the Cocoa Research Institute of Ghana and Yayra Glover Limited, a licensed organic cocoa producing and marketing company in Ghana. Whilst 140 conventional cocoa bean samples were collected from the seven cocoa-producing regions of Ghana under the guide of the Quality Control Company and Cocoa Marketing Company of COCOBOD. The two categories of cocoa beans (organic and conventional) according to the producers were fermented for 6 days using heap protocols similar to those described by other authors [25]. The cocoa bean samples were well labelled and transported in marked jute bags to the Department of Agricultural Engineering Research Laboratory, University of Cape Coast, for further examination. Spectral measurements were taken on the whole cocoa beans, whilst chemical examinations were conducted on the ground samples.

### 2.2. Sample Spectral Measurement

The handheld NIR spectrometer (Tellspec^®^) was used to take the spectrum of each cocoa bean sample in an absorbance unit (log 1/*R*); *R* = reflectance. The NIR spectroscopic dataset was developed in a wavelength range of 900-1700 nm. The instrument was operated using a smartphone application, and spectroscopic data stored in the cloud remotely was downloaded onto the laptop. All the cocoa bean samples were scanned three times in a transparent polythene bag at different sides, and the spectrum for each sample was the mean of the three scans. Scanning of the samples was carried out at an ambient temperature of 25 ± 1°C with a humidity of 60%. The spectra were downloaded with permission from Tellspec Ltd.

### 2.3. Software Tool

All preprocessing and analysis of the spectra data were performed using multivariate analysis software in MATLAB version 9.6.0 (The MathWorks Inc., USA) with windows 10 Pro software package for data treatment.

### 2.4. Dataset Partitioning

The spectroscopic datasets obtained from 260 samples of organic and conventional cocoa beans were preprocessed with appropriate techniques. The spectral data obtained from the samples were randomly divided into two different datasets called: calibration set (spectroscopic data from 182 samples) and prediction set (spectroscopic data from 78 samples). The calibration set which represented 70% of the data was used to construct the models, whereas the remaining 30% of the data were used for the prediction set which was used to evaluate the predictive capability of the built models.

### 2.5. Spectral Preprocessing Approaches

**Figure 1:**
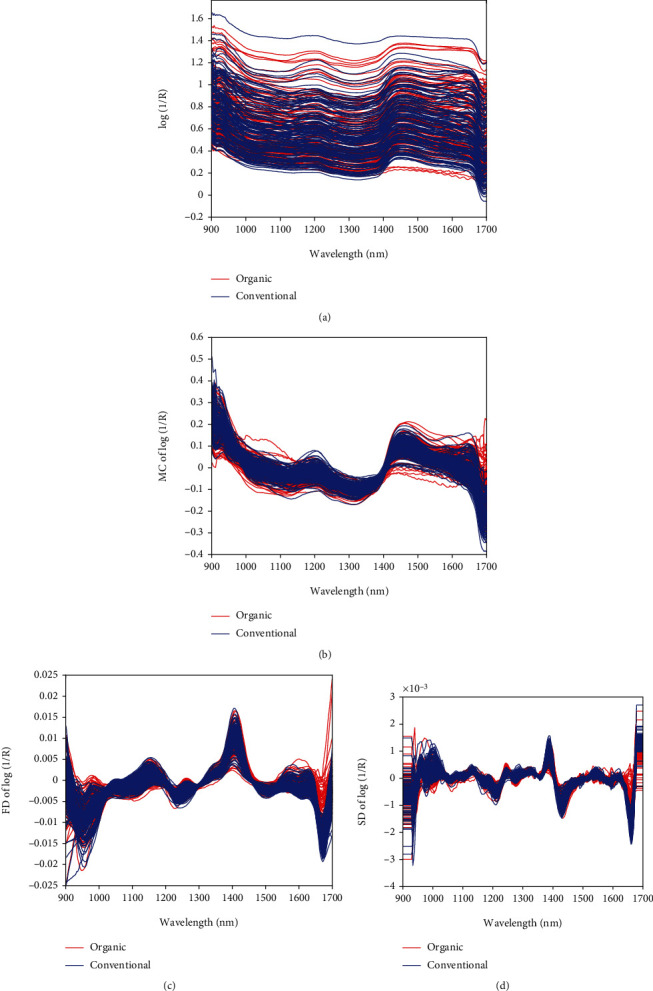
Spectra: (a) raw spectra, (b) mean centering, (c) first derivative, and (d) second derivative of cocoa bean samples.

The raw NIR spectra as shown in Figure 1(a) contain unwanted, beneficial, and nonuseful information of the cocoa bean samples. This could be as a result of interferences from the scattering of light from the samples, spectra poor reproducibility, temperature variations, and or background noises [26]. Therefore, chemometric pretreatment of the dataset has to acquire only the useful properties of samples, whilst keeping the similarities and variations among the primary observations was adopted. To accomplish this, three spectral preprocessing approaches such as MC (mean centering), FD (first derivative), and SD (second derivative) were comparatively employed in MATLAB version 9.6.0 as shown in Figures 1(b)–1(d). MC is a spectral preprocessing approach carried out by computing the mean spectrum of the dataset and deducting the mean from each spectrum [27]. FD preprocessing approach which is assessed as the difference between two consequent spectra measurement points eliminates baseline effects. SD transformation algorithm is employed in the separation of overlapped peaks, resolution enhancement, removal of additive, and multiplicative baseline in the spectra. Before the application of the SD preprocessing technique, the NIR spectra were smoothed using the Savitzky-Golay algorithm [28]. Generally, the Savitzky-Golay smoothing SD algorithm best improved the linearity and corrected offset in NIR data.

### 2.6. Principal Component Analysis (PCA)

Furthermore, the principal component analysis (PCA) was deployed on all the preprocessed NIR datasets to identify any cluster trend (to detect probable groupings). The PCA has been an unsupervised pattern recognition algorithm that extracted information from correlated matrices to see probable data leanings in a dimensional scatter plot. In the PCA analysis, the datasets coupled with spectra were converted into a small number of uncorrelated but explainable variables referred to as principal components (PCs). Similar samples congregated closer to each other and vice versa. The graphic profile of PCA results yielded initial output for the determination of possible variations and resemblances in a dataset. Usually, PC1, PC2, PC3, PC4, PC5, etc. explain and give relevant information in descending order [29].

### 2.7. Multivariate Classification Algorithms

#### 2.7.1. RF

RF (random forest) is an ensemble procedure which is based on tree classifiers. It grows many classification trees in order to produce accurate discrimination. In RF, each tree grows on an independent bootstrap sample obtained from the calibration sample/data [30]. Classification of the new feature vector is achieved by classifying the input vector with each of the trees in the forest. A classification is given by each tree, often considered as that tree's vote for that class. The forest selects the classification with the maximum votes over all the trees in the forest [31]. RF computations comprise two measures of variable importance (based on rough-and-ready measure and permutations) and measures of the resemblance of data points that could be applied for graphical representation, multidimensional scaling, imputing missing values, and clustering [32].

#### 2.7.2. KNN

KNN (*K*-nearest neighbours) is a nonparametric and linear learning algorithm where the distance between each of the samples of the calibration set and unknown sample is assessed; for more information, refer to Reference [33]. For KNN approach, the parameter *K* has a huge influence on the classification rate of the KNN model. The selection of *K* was optimised by computing the calibration ability with a preferably an old number of small *K* values. In this study, PCs were applied as an input data in KNN model. KNN model efficiency was examined by the number of parameter *K* and PCs [34].

#### 2.7.3. LDA

LDA (linear discriminant analysis) is a linear and parametric supervised pattern recognition approach mostly applied to discover a linear combination of features, and the resultant combination may be employed as a linear classifier. LDA concept is founded on the determination of linear discrimination functions that produce the ratio between-class variance and decrease the ratio of within-class variance. In the LDA approach, the classes are linearly separated and keep to a normal distribution [35]. Also, the LDA is viewed as PCA in which the number of PC (principal component) is key to the performance of the LDA classification model.

#### 2.7.4. PLS-DA

PLS-DA (partial least squares-discriminant analysis) is a linear differentiation technique that combines properties of partial least squares regression with the discrimination presentation of a differentiation technique [36]. The PLS-DA with a *k*-fold cross-validation was deployed to screen out and differentiate organic cocoa beans from conventional ones and to prevent overfitting of the calibration models. This qualitative transformational technique was performed to extract principal components from the spectral information, decrease the number of variables employed in the model, and find which variables carry the class separating information by rotating principal component analysis (PCA). This combines the variables in the dataset to calculate factors that maximize the correlation value with the different classes [37]. PLS-DA concurrently decomposes spectral and class matrices and extracts the spectral data most associated with the classes that can lead to the development of a reliable and accurate identification model [38].

### 2.8. Performance Assessment of Multivariate Data Analysis Algorithms

Qualitatively, the performance of the PLS-DA classification model was assessed according to identification rate or accuracy, sensitivity, specificity, and efficiency. Accuracy is the proportion of samples, either organic cocoa beans or conventional cocoa beans correctly identified in a population, either in the calibration set or prediction set. It computes the degree of closeness or veracity of the measured result to the true value or analytical sample. Sensitivity evaluates the capability of the model to correctly identify and classify samples belonging to the targeted class (i.e., organic cocoa bean class). It measures how good the model is at detecting and classifying an organic cocoa bean from a conventional cocoa bean. Specificity evaluates the capability of the model to correctly detect and reject samples that belong to both classes (i.e., organic cocoa bean class and conventional cocoa bean class). It assesses how likely conventional cocoa beans could be ruled out correctly from organic cocoa beans. Efficiency is defined as the geometric mean of sensitivity and specificity in both calibration and prediction sets. Sensitivity and specificity depend on the values of true positive (TP), true negative (TN), false positive (FP), and false negative (FN) ([39]; Wang et al. [40]). The assessment of the model's performance was done according to the methods described by Chen et al. [41] and Zhang et al. [42]. Computation of these parameters was done using Equations (1)–(4).

(1) $$\begin{array}{c} \text{Accuracy}=\frac{\text{TN}+\text{TP}}{\text{TN}+\text{TP}+\text{FN}+\text{FP}}, \end{array}$$

(2) $$\begin{array}{c} \text{Sensitivity}=\frac{\text{TP}}{\text{TP}+\text{FN}}, \end{array}$$

(3) $$\begin{array}{c} \text{Specificity}=\frac{\text{TN}}{\text{TN}+\text{FP}}, \end{array}$$

(4) $$\begin{array}{c} \text{Efficiency}=\sqrt{\text{Sensitivity}*\text{Specificity}}. \end{array}$$

## 3. Results

### 3.1. Cocoa Compositional Quality Characteristics

The compositional quality characteristics varied according to the cocoa category as presented in Table 1. For both categories of cocoa beans, crude fat was the major constituent. These findings are consistent with results obtained by [18]. Crude fat, total carbohydrate, total polyphenols, total flavonoid, and antioxidant contents showed statistical differences between organic cocoa and conventional cocoa samples (*p* > 0.05). There were no significant statistical differences at *p* > 0.05 between the two cocoa bean categories for crude fibre and protein, although their obtained values numerically differed.

### 3.2. NIR Spectra Examination

**Figure 2:**
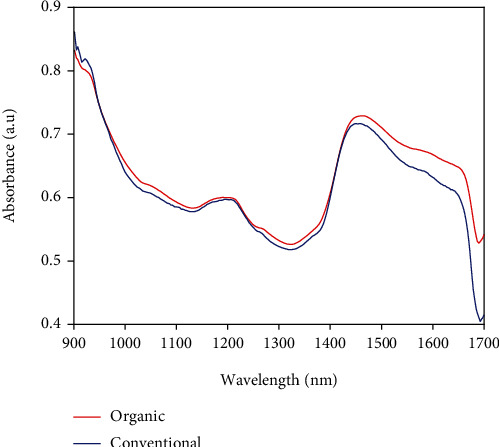
Mean spectra profile of organic and conventional cocoa bean samples.

The raw spectra of the 260 cocoa bean samples obtained in the wavelength range of 900 to 1700 nm are presented in Figure 1(a). This spectral wavelength range can offer useful features for the differentiation of organic and conventional cocoa bean samples, though the raw spectra profile seemed to be similar. There was a wide variation of baseline shift in the spectra due to background information, particle size effect, temperature variation, and noise [26]. This made it difficult to determine exact bands in the original spectra due to the high degree of overlapping of bands. Hence, chemometric preprocessing analysis of the dataset was applied to acquire only the useful properties of samples and build a reliable model whereas keeping the similarities and variations among the primary observations. Among the chemometric analyses applied, the SD preprocessing approach best smoothens the original spectra and eventually leads to satisfactory classification, and its spectra are presented in Figure 1(d). This contributed to clear and noticeable groupings as shown in the mean spectra profile in Figure 2. It depicts specific absorption bands observed from main valleys and peaks that are related to vibrations of chemical bonds such as N-H, S-H, C=O, -CH_3_, and CH_2_ ([43–45]; Zhang et al. [46]). These chemical bond vibrations are associated with major biochemical constituents such as polyphenols, flavonoids, alkaloids, antioxidants, volatile and nonvolatile acid, fats, proteins, carbonyl group, C-H deformation and C-H stretch as seen in Table 2, and other composites present in the cocoa beans no matter the production method or origin. Specifically, the absorption band attributable to C-H bond of cocoa that is mainly connected to proteins and fats was found around 910 nm [13]. Absorption bands of 1000-1100 nm are attributable to C-H stretch 1^st^ overtone, carbonyl groups (-CH_2_, CH_3_-, and -CH=CH-) [13, 28, 47]. An observable absorption band around 1440 nm might be associated with 1^st^ overtone of starch, moisture, and sugars [13]. These spectral wavelength bands might have significantly contributed to the classification of organic and conventional cocoa beans.

### 3.3. Spectral Presentation and Principal Component Analysis (PCA)

**Figure 3:**
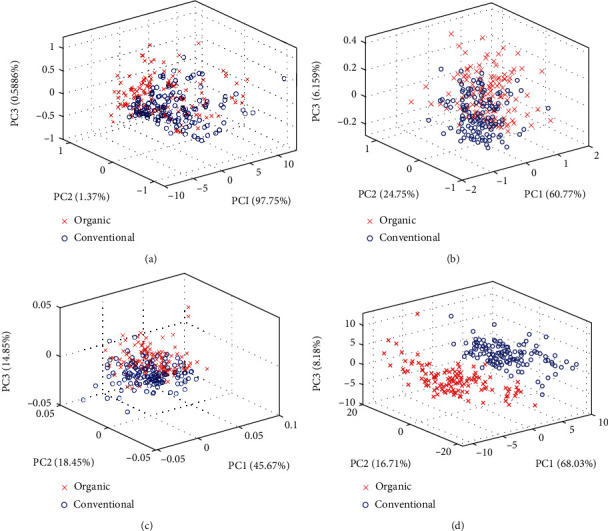
PCA score plot of the first three PCs of organic and conventional cocoa beans: (a) raw, (b) MC, (c) FD, and (d) SD.

To observe a visible trend of the samples and evaluate the relations among samples, PCA was performed using the raw spectral data and the outcomes presented in Figures 3(a)–3(d). The PCA after second derivative preprocessing yielded a good cluster trend. The three topmost PCs extracted from the 260 samples were PC1 (68.03%), PC2 (16.71%), and PC3 (8.18%). It shows that the three topmost PCs can explain 92.92% of the variance information from the spectra dataset that covers the relevant biochemical information in the samples. PCA technique brings out useful relevant information and removes irrelevant ones so that bean samples with the same characteristics are clustered nearer to each other. Thus, the graphic output could be used to discover the variances between the categories of cocoa bean samples used.  Figure 3(d) depicts that two main groups of cocoa bean samples were used in the study. The groups cover a broader array of cocoa beans. The graphic plot offers relevant information that could be used for the determination of differences between organic and conventional cocoa bean samples. PCA is not a classification tool but it showed the data trend in visualizing dimension space [48].

**Figure 4:**
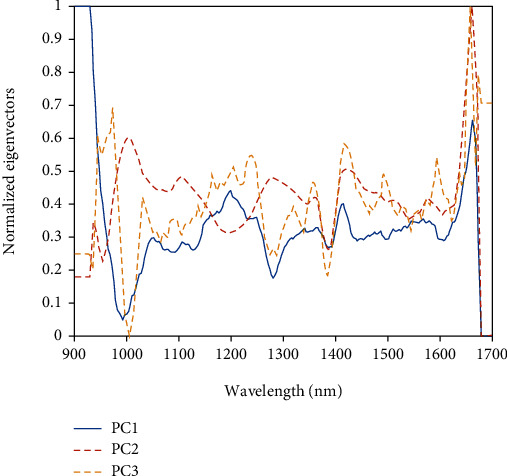
PCA loadings of the top three latent variables of cocoa bean samples.

PCA loadings as shown in Figure 4 were performed to give an explanation as to how much each wavelength contributed to the significant variation in the data. It was observed that wavelengths corresponding to the biggest eigenvector loading values for PC1 (68.03%) were situated around the range of 986 nm associated with pH 1^st^ overtone absorption peak O-H stretching (Wang et al. [49]) and O-H stretch 2^nd^ overtone of carbohydrate; 1280 nm and 1417 nm are 2^nd^ overtone bands C-H bond stretching and C-H combination (aromatic), respectively. The peak around 1200 nm could be attributed to the 1^st^ overtone of C-H stretch [50]. These absorption bands are characteristics of proteins, fats, and aromatic compounds found in cocoa beans [45, 51, 52]. Observable absorption band around 1608 nm might be ascribed to 1^st^ overtone of C-H stretching (Zhang et al. [53]). PC2 explains 16.71% of the variance, and the biggest vibration placed around 958 nm, 973, and 1395 nm correlated with 2^nd^ overtone of OH stretch of carbohydrate, 2^nd^ overtone of N-H stretching of fat, and 2x C-H stretch+C-H deformation of protein, respectively (Zhang et al. [53]). PC 3 explains 8.18% of the variation, and it appeared to be the mirror image of the cocoa bean spectra of the cocoa bean, and this accounted for the slight differences in particle size. The differences correlated to compositional variations among the cocoa bean categories. It implied a particular chemical constituent alone or in combination with others contributed the largest influence that explained the basis for the detected variations between the cocoa bean samples.

### 3.4. Performance of Classification Models

A qualitative analysis such as RF, KNN, LDA, and PLS-DA was performed as the PCA was not able to accurately classify the samples according to organic and conventional cocoa beans. The results from different classification models for the discrimination of organic and conventional cocoa beans are reported in Table 3. Every multivariate classification algorithm has its potentials and limitations. As shown in Table 3, the SD processing (17-point window, 2^nd^-order polynomial) highly enhanced the performance of all the multivariate classification algorithms in both the calibration set and prediction set than MC and FD.

#### 3.4.1. RF

The *k*-fold cross-validation results showed that the RF algorithm with 9 PCs on normalized data provided correct identification rates with 96.09% and 98.37% efficiency in the calibration set and prediction set, respectively (Table 3). The optimum number of PCs was based on the best classification rate performed by *k*-fold cross-validation. In Table 3, the best classification rate by RF model for calibration set was 96.15% and 98.08% for the prediction set at an optimum number of 9 PCs.

#### 3.4.2. KNN

In Table 3, the *k*-fold cross-validation outcomes disclosed that the KNN algorithm with PCs equals 5 on normalized data provided a correct classification rate with 91.49% efficiency for the calibration set and 92.79% for the prediction set. Table 3 demonstrates that the best classification rate for the calibration set was 91.35% and 92.31% for the prediction set.

#### 3.4.3. LDA

In Table 3, the *k*-fold cross-validation results showed that the LDA algorithm with PCs equals 5 on normalized data provided a correct classification rate with 90.38% calibration set efficiency and 98.06% prediction set efficiency. Table 3 demonstrates that the best classification rate for the calibration set was 90.38% and 98.08% for the prediction set.

#### 3.4.4. PLS-DA Model

**Figure 5:**
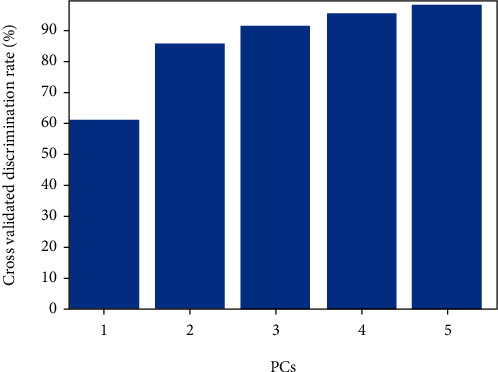
The *k*-fold cross-validation discrimination rates of second derivative preprocessed PLS-DA models at 5 PCs.

Table 3 shows the performance of the PLS-DA classification algorithm used in identifying organic and conventional cocoa beans. *k*-fold cross-validation outcomes demonstrated that the PLS-DA technique with 5 principal components (PCs) on normalized data provided correct identification rates with 100% efficiency in the prediction set.  Figure 5 displays the performance of the PLS-DA model for solving the discrimination problems after *k*-fold cross-validation. The optimum number of PCs was based on the best classification accuracy achieved by *k*-fold cross-validation. In Table 3, the best classification rate for both calibration set and prediction set was 100.00% at an optimum number of 5 PCs.

### 3.5. Overall Performance of Classification Algorithms

The identification rates of multivariate classification algorithms are presented in Table 4. In this table, we compare the classification accuracy of the RF, KNN, LDA, and PLS-DA models. Comparatively, the results show that the performance of the PLS-DA established model was superior to others, viz., RF, KNN, and LDA (Table 4). The result is in agreement with that of [56] where the PLS-DA technique performed better in the identification of sorghum cultivars. The discrimination stability for all the cocoa bean samples investigated increased in the order of KNN < LDA < RF < PLS-DA denoted by identification rate.

### 3.6. Discussion

There were observed differences in chemical compositions of organic cocoa beans and conventional ones (as seen in Table 1). These could largely be attributed to the influence of production methods and partly to the reaction of inherent compositions of the organic and conventional cocoa beans to the fermentation process (which was carried out using the same protocols for both categories of cocoa beans). Chocolate flavour compounds do not only originate by character precursor formation during fermentation but could also be generated during production management systems [57]. Thus, the composition of organic and conventional cocoa beans interacted with the fermentation process in the formation of cocoa flavour quality constituents. The use of synthetic fertilizers and chemicals in conventional method contributed to variations in the cocoa bean biochemical composition that could lead to a distinct cluster trend.

The spectra obtained from scanning of the organic and conventional cocoa bean samples with the handheld NIR spectrometer produced a spectral profile that displayed multiple wavelength bands and peaks as shown in Figure 1(d). The bands consisted of overtones and combinations of fundamental vibrations that matched the chemical compositions which provided exclusive fingerprint of the cocoa bean categories employed in this study. The preprocessing of the spectra profile into mean was performed, and there were two groupings representing the two distinct cocoa bean samples used as seen in Figure 2. This is due to the unique biochemical and physical properties of each bean group to give a well-defined separation trend.

The comparative analysis of the PCA cluster using different preprocessing techniques revealed that the second derivative treatment performed better by showing a clear cluster trend as shown in Figure 3. The clustering can be explained by the biochemical compositions in each of the cocoa bean samples as a result of differences in the categories of the cocoa bean either been organic or conventionally produced cocoa bean. The contributions of the three topmost PCs were 92.92% for the total variance in the original data. Nevertheless, PCA does not give definite identification because it is not a classification tool; however, it preserves much variance in a high-dimensional space by reducing dimensionality. PCA loading plot in Figure 4 shows the most important wavelength bands which contributed to the cluster trend of the cocoa bean samples and were located at around 986, 1200, 1280, 1417, and 1068 nm for PC1; 958, 973, 1395, and 1460 nm for PC2; and 1005, 1440, and 1483 nm for PC3. The wavelengths at 958, 973, 986, 1440, and 1460 nm are due to 1^st^ overtone and 2^nd^ overtone of O-H/O-H stretch; 1005 and 1483 nm are attributable to 2^nd^ overtones of N-H stretch; 1200 and 1608 nm are related to C-O from COOH typical and 1^st^ overtone of C-H stretch; 1280 nm band might be characterised by 2^nd^ overtone bands C-H bond stretching; 1375 and 1417 nm could be associated with C-H vibration modes; 1395 nm and 1417 nm absorption band might correspond to 2x C-H stretch+C-H deformation and combination. These observable wavelengths are principally characterised by the asymmetric stretching, overtones, and combinations of vibrations of C-H, N-H, O-H, and C=O which are triggered by constituents such as fats, water, polyphenols, fibre, organic acids, alkaloids, polysaccharides, amines, and aromatic compounds found in cocoa beans ([43–45]; Zhang et al. [53]).  Table 2 gives additional information on the observable absorption bands and their associated chemical constituents. These spectra observations echoed the outcome of the chemical compositions of the two categories of cocoa beans studied. These spectral wavelength bands might have significantly contributed to the classification of organic and conventional cocoa beans as seen in Table 3.

Four other pattern recognition algorithms which are known to have potentials in solving identification problems were applied. The pattern recognition algorithms such as RF, KNN, LDA, and PLS-DA were applied to build a classification model and to ensure their stability cross-validation was done. PLS-DA model produced classification accuracy of 100.00% in both the calibration set and prediction set, whereas the classification accuracies for the calibration set and prediction set were 96.15% and 98.08% for RF, 91.35% and 92.31% for KNN, and 90.38% and 98.08% for LDA (Table 4). The experimental outcomes showed that the PLS-DA algorithm was superior to RF, KNN, and LDA algorithms. This can be due to the fact that the PLS-DA algorithm possesses stronger and added potential of self-adjusting and self-learning properties. For cocoa bean categories used in this work, the biochemical compositions and complex organoleptic properties can explain why RF, KNN, and LDA could not deliver the optimum solution. The PLS-DA delivered its best performance at 5 PCs. High number of PCs as seen in the RF model may result in low generalization in the performance lowering the efficiency of its model.

Generally, the optimum classification accuracy (100%) received could largely be attributed to the influence of production methods and partly to the reaction of inherent compositions of the organic and conventional cocoa beans to the fermentation. Cocoa bean flavour compounds do not only originate by character precursor formation during fermentation but could also be generated during production management systems [57]. Thus, the composition of organic and conventional cocoa beans interacted with the fermentation process in the formation of cocoa flavour quality constituents. The use of synthetic fertilizers and chemicals in the conventional method contributed to variations in the cocoa bean biochemical composition leading to the distinct cluster trends and differentiation of the cocoa samples used in this experiment. Also, according to other authors, organically produced foods show high polyphenols and ascorbic acid contents as a response to stress stimuli [58]. More so, organic crops often grow more slowly compared to synthetic fertilized crops with readily available mineral nutrients and this might reduce their water content leading to a higher concentration of some plant compounds [59]. It is therefore expected that organically produced cocoa will have higher concentrations of some compounds (polyphenols, protein, carbohydrate, fibre, and total flavonoids) as recorded in this study. This phenomenon might have contributed to the accurate classification of the different categories of cocoa beans used in this study by the handheld NIR spectroscopy.

## 4. Conclusions

This work represents the first study to successfully evaluate the application of a low-cost handheld NIR spectrometer and chemometric classification techniques for rapid nondestructive screening and authentication of organic and conventional cocoa beans produced in Ghana. The PCA score plot exhibited the feasibility of identifying cocoa bean categories. Four different chemometric classification algorithms, viz., RF, KNN, LDA, and PLS-DA, were comparatively performed for the construction of classification models. PLS-DA exhibited superior performance over the others (RF, KNN, and LDA) after second derivative (SD) preprocessing for the differentiation of organic cocoa beans from conventional ones. PLS-DA model yielded classification accuracy of 100% in both calibration set and prediction set. The application of handheld NIR spectrometer and PLS-DA algorithms could be employed as a simple, on-site, cost-effective, rapid, and ecofriendly technique for accurate identification of organic cocoa beans and conventional ones to prevent fraud and ensure the integrity of organic cocoa beans.

## Figures and Tables

**Table 1:** Proximate compositions (g/100 g) and phytonutrient contents for organic and conventional cocoa samples (mean ± SD).

Samples	Crude fat (%)	Crude fibre (%)	Crude protein (%)	Total carbohydrate (%)	Total polyphenol content (mgGAE/g)	Total flavonoid content (mgQE/g)	Total antioxidant capacity (mgAEAC/g)
Organic	41.75 ± 0.20^b^	7.83 ± 0.08^a^	15.77 ± 0.31^a^	25.63 ± 0.46^a^	61.66 ± 0.16^a^	81.44 ± 0.56^a^	108.78 ± 0.45^b^
Conventional	43.66 ± 0.15^a^	7.56 ± 0.07^a^	15.45 ± 0.24^a^	22.41 ± 0.45^b^	59.73 ± 0.22^b^	72.66 ± 0.39^b^	130.03 ± 0.56^a^

NB: same letters show that there is no statistical difference (*p* > 0.05) among samples. GAE: gallic acid equivalent; QE: quercetin equivalent; AEAC: ascorbic acid equivalent antioxidant content.

**Table 2:** NIR wavelength band assignments.

Wavelength (nm)	Functional group	Band assignments
958	OH	O-H 2^nd^ overtone stretch of carbohydrate [54]
973, 1005	NH_3_	N-H 2^nd^ overtone stretch associated with fat (Chu [53])
986	O-H	O-H 1^st^ and 2^nd^ overtones stretch of absorption peak of starch [54]
1200	C-H	C-H 1^st^ overtones stretch related to proteins and starch [50]
1280	C-H	C-H 1^st^ overtone bond stretching corresponding to fats and aromatic compounds [52]
1483	O-H	O-H 2^nd^ overtone stretch corresponding moisture (Zhang et al. [53])
1417	O-H	H_2_O band groups corresponding to weakly bounded water and aromatic compounds [43]
1440, 1460	O-H	O-H stretch 1st overtone of starch, water band, and sugars ([13]; Zhang et al. [46])
1608	C=O	C-O from COOH typical of amines and acidity [55]

**Table 3:** Classification models with preprocessing algorithms for cocoa bean samples.

Models	Preprocessing	PCs	Evaluation	Performance (%)	
				Calibration set	Prediction set
RF	Raw	3	Accuracy	67.79	67.31
			Sensitivity	69.23	69.23
			Specificity	66.35	65.38
			Efficiency	67.77	67.28
	MC	7	Accuracy	85.58	88.46
			Sensitivity	82.69	84.62
			Specificity	88.46	92.31
			Efficiency	85.53	88.38
	FD	7	Accuracy	89.90	88.46
			Sensitivity	90.65	91.30
			Specificity	89.11	86.21
			Efficiency	89.88	88.72
	SD	9	Accuracy	96.15	98.08
			Sensitivity	94.95	96.77
			Specificity	97.25	100.00
			Efficiency	96.09	98.37
KNN	Raw	3	Accuracy	65.38%	69.23
			Sensitivity	71.29%	62.07
			Specificity	59.81%	78.26
			Efficiency	65.30%	69.70
	MC	5	Accuracy	79.81	82.69
			Sensitivity	82.86	76.00
			Specificity	76.70	88.89
			Efficiency	79.72	82.19
	FD	5	Accuracy	80.77	80.77
			Sensitivity	75.76	83.87
			Specificity	85.32	76.19
			Efficiency	80.40	79.94
	SD	5	Accuracy	91.35	92.31
			Sensitivity	87.27	95.00
			Specificity	95.92	90.63
			Efficiency	91.49	92.79
LDA	Raw	3	Accuracy	69.71	69.23
			Sensitivity	72.38	64.00
			Specificity	66.99	74.07
			Efficiency	69.63	68.85
	MC	5	Accuracy	84.13	78.85
			Sensitivity	86.14	86.21
			Specificity	82.24	69.57
			Efficiency	84.17	77.44
	FD	5	Accuracy	78.85	80.77
			Sensitivity	83.65	88.46
			Specificity	74.04	73.08
			Efficiency	78.70	80.40
	SD	5	Accuracy	90.38	98.08
			Sensitivity	89.42	100.00
			Specificity	91.35	96.15
			Efficiency	90.38	98.06
PLS-DA	Raw	3	Accuracy	77.88	78.85
			Sensitivity	80.81	83.87
			Specificity	75.23	71.43
			Efficiency	77.97	77.40
	MC	5	Accuracy	92.79	94.23
			Sensitivity	96.97	90.32
			Specificity	88.99	100.00
			Efficiency	92.89	95.04
	FD	5	Accuracy	98.56	100.00
			Sensitivity	99.04	100.00
			Specificity	98.08	100.00
			Efficiency	98.56	100.00
	SD	5	Accuracy	100.00	100.00
			Sensitivity	100.00	100.00
			Specificity	100.00	100.00
			Efficiency	100.00	100.00

**Table 4:** Overall performance of classification algorithms.

Models	Total cocoa bean samples		PCs	Identification rate (%)	
	Calibration set	Prediction set		Calibration set	Prediction set
RF	182	78	9	96.15	98.08
KNN	182	78	5	91.35	92.31
LDA	182	78	5	90.38	98.08
PLS-DA	182	78	5	100.00	100.00

## Data Availability

The dataset used is available from the corresponding author upon judicious request.
